# P-2150. Single Centre Real World Experience of Evaluating Cytomegalovirus Cell Mediated Immunity using an ELISpot Assay in Solid Organ Transplant Recipients: Helpful or Not?

**DOI:** 10.1093/ofid/ofaf695.2313

**Published:** 2026-01-11

**Authors:** Min Ru Chee, Jarett Vanz-Brian Pereira, Carolyn Shan-Yeu Tien, Ian Tatt Liew, Sobhana D/O Thangaraju, Terence Kee, Soon Hwee Ng, Thuan Tong Tan, Ban Hock Tan, Wei Yee Wan, Shimin Jasmine Chung

**Affiliations:** Singapore General Hospital, Singapore, Not Applicable, Singapore; Singapore General Hospital, Singapore, Not Applicable, Singapore; Singapore General Hospital, Singapore, Not Applicable, Singapore; Singapore General Hospital, Singapore, Not Applicable, Singapore; Singapore General Hospital, Singapore, Not Applicable, Singapore; Singapore General Hospital, SingHealth Duke-NUS Transplant Centre, Singapore, Singapore, Not Applicable, Singapore; Singapore General Hospital, Singapore, Not Applicable, Singapore; Singapore General Hospital, Singapore, Not Applicable, Singapore; Singapore General Hospital, Singapore, Not Applicable, Singapore; Singapore General Hospital, Singapore, Not Applicable, Singapore; Singapore General Hospital, Singapore, Not Applicable, Singapore

## Abstract

**Background:**

Cytomegalovirus (CMV) infection can negatively impact outcomes in solid organ transplantation (SOT). In addition to CMV sero-evaluation, CMV specific cell-mediated immunity (CMV-CMI) to guide CMV prevention and management is now gaining traction. We aim to assess the real world use of CMV-CMI in SOT at our centre.

**Table 1:**
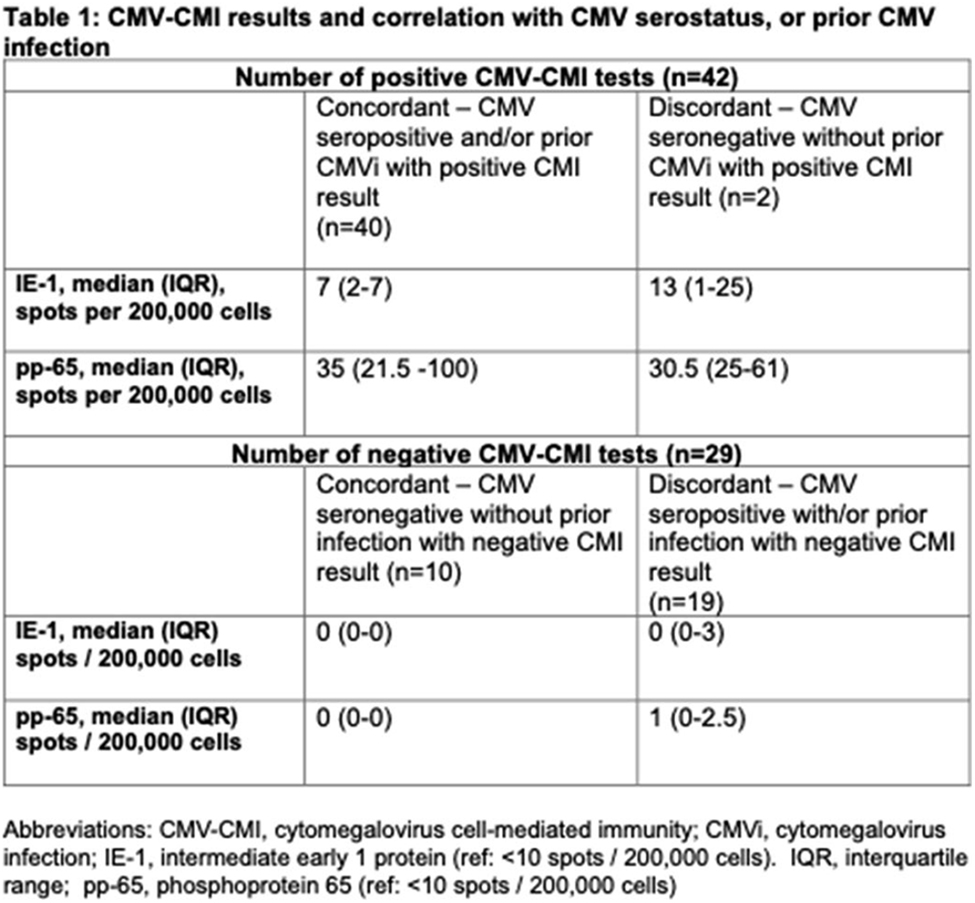
CMV-CMI results and correlation with CMV serostatus, or prior CMV infection

**Table 2:**
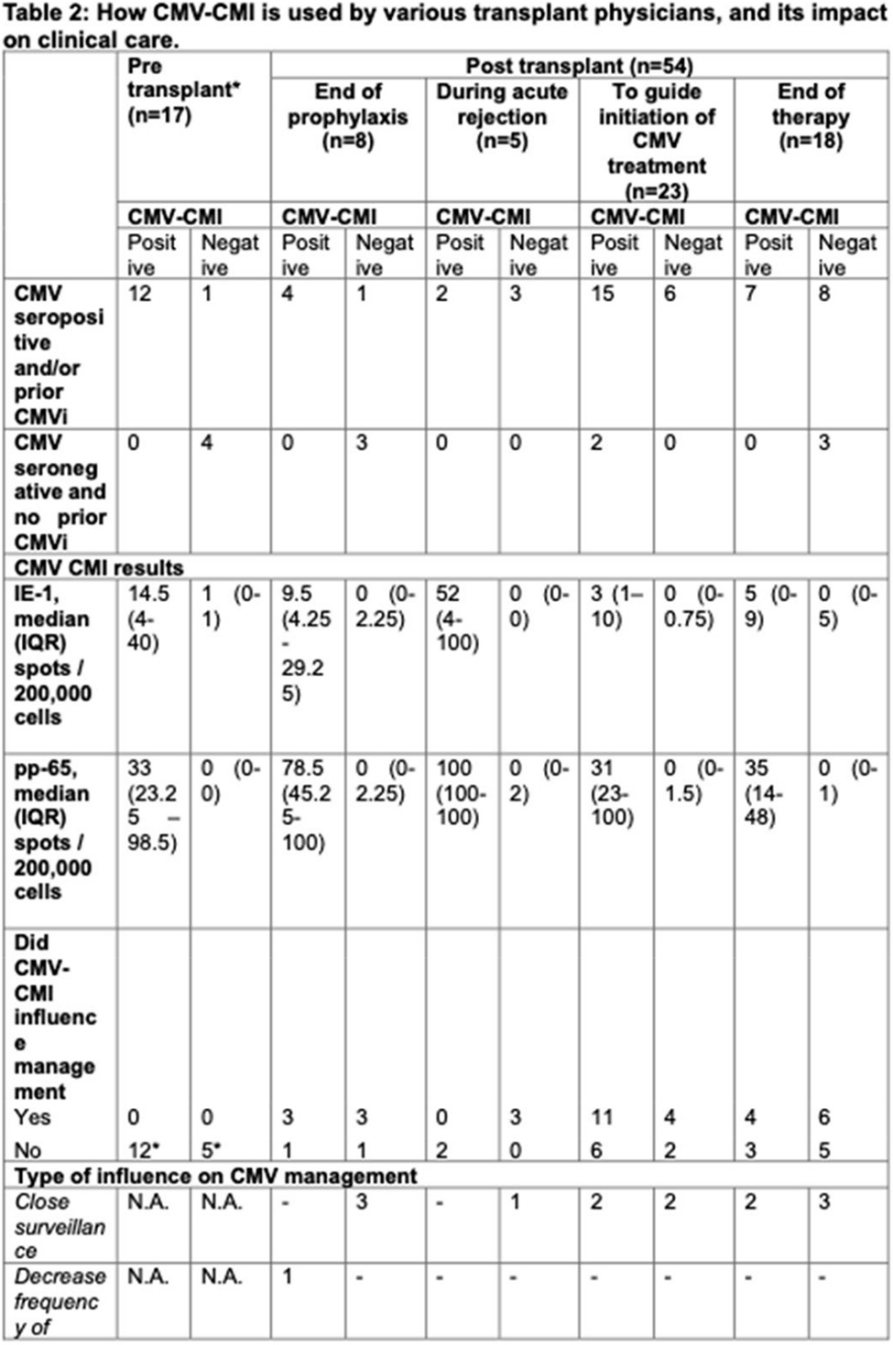
How CMV-CMI is used by various transplant physicians, and its impact on clinical care.

**Methods:**

This is a single-centre prospective observational study to study the role of CMV-CMI using the ELISpot CMV assay to guide CMV management in SOT. Recipients with CMV-CMI tested between Mar ‘24 and Feb ‘25 were included.

**Results:**

Eighty-four CMV-CMI assays were performed in 65 unique SOT recipients [30 (46.2%) females, 35 (53.8%) males]. Seventy-one assays were suitable for analysis; 8 were rejected due to inadequate peripheral blood mononuclear cells, while 5 were inconclusive. These assays were conducted in patients with kidney (n=61), heart (n=3), liver (n=4) and uterine (n=3) transplants; 17 were for pre-transplant evaluation, 8 at the end of prophylaxis, 5 during acute rejection, 23 to guide initiation of CMV treatment and 18 at the end of treatment. The patients’ median age was 54 (IQR 19-73) years. Among 42 positive CMI results, 40/42 (95.2%) were concordant with CMV seropositivity or prior CMV infection. Twenty-nine patients had a negative CMV-CMI; 19/29 (65%) had discordant results. CMV prophylaxis was protocol-driven, not CMV-CMI guided. However, CMV-CMI guided post-prophylaxis strategies in 6/8 (75%) patients. In acute rejection, negative CMV-CMI prompted initiation of prophylaxis or close surveillance. CMV-CMI testing was often used to guide treatment initiation; treatment was deferred with positive CMV-CMI (7/15), while a negative test prompted early treatment or closer surveillance (4/6). After CMV treatment, CMV-CMI informed post-treatment strategies in 10/18 (55.6%) cases; 6 with negative CMV-CMI had close CMV surveillance, initiation of secondary prophylaxis, and/or reduced immunosuppression, while 4 with a positive test had prophylaxis withheld.

**Conclusion:**

CMV-CMI influenced decisions on CMV management after SOT although no standardised protocols have yet been implemented. CMV-CMI cut-offs and its utility to guide CMV management could be evaluated and defined in larger cohorts / clinical trials.

**Disclosures:**

All Authors: No reported disclosures

